# Ploidy of Cell-Sorted Trophic and Cystic Forms of *Pneumocystis carinii*

**DOI:** 10.1371/journal.pone.0020935

**Published:** 2011-06-14

**Authors:** Anna Martinez, El Moukhtar Aliouat, Annie Standaert-Vitse, Elisabeth Werkmeister, Muriel Pottier, Claire Pinçon, Eduardo Dei-Cas, Cécile-Marie Aliouat-Denis

**Affiliations:** 1 Université Lille Nord de France, Lille, France; 2 INSERM U1019, CNRS UMR 8204, Center for Infection and Immunity of Lille (CIIL), Lille, France; 3 Institut Pasteur de Lille, Lille, France; 4 UDSL (Université Droit et Santé de Lille), Lille, France; 5 MICPaL Facility, IFR142, CNRS UMR 8161, Lille, France; 6 EA2694, Department of Biostatistics, Lille, France; 7 Regional and University Hospital Center (CHULille), Biology & Pathology Center (CBP), Parasitology-Mycology, Lille, France; Institut National de la Santé et de la Recherche Médicale - Institut Cochin, France

## Abstract

Once regarded as an AIDS-defining illness, *Pneumocystis* pneumonia (PcP) is nowadays prevailing in immunocompromised HIV-negative individuals such as patients receiving immunosuppressive therapies or affected by primary immunodeficiency. Moreover, *Pneumocystis* clinical spectrum is broadening to non-severely-immunocompromised subjects who could be colonized by the fungus while remaining asymptomatic for PcP, thus being able to transmit the infection by airborne route to susceptible hosts. Although the taxonomical position of the *Pneumocystis* genus has been clarified, several aspects of its life cycle remain elusive such as its mode of proliferation within the alveolus or its ploidy level. As no long-term culture model exists to grow *Pneumocystis* organisms *in vitro*, an option was to use a model of immunosuppressed rat infected with *Pneumocystis carinii* and sort life cycle stage fractions using a high-through-put cytometer. Subsequently, ploidy levels of the *P. carinii* trophic and cystic form fractions were measured by flow cytometry. In the cystic form, eight contents of DNA were measured thus strengthening the fact that each mature cyst contains eight haploid spores. Following release, each spore evolves into a trophic form. The majority of the trophic form fraction was haploid in our study. Some less abundant trophic forms displayed two contents of DNA indicating that they could undergo (i) mating/fusion leading to a diploid status or (ii) asexual mitotic division or (iii) both. Even less abundant trophic forms with four contents of DNA were suggestive of mitotic divisions occurring following mating in diploid trophic forms. Of interest, was the presence of trophic forms with three contents of DNA, an unusual finding that could be related to asymmetrical mitotic divisions occurring in other fungal species to create genetic diversity at lower energetic expenses than mating. Overall, ploidy data of *P. carinii* life cycle stages shed new light on the complexity of its modes of proliferation.

## Introduction

The *Pneumocystis* genus comprises diverse species of micro-mycetes that proliferate in the alveolus of a wide variety of mammalian hosts. In immunocompromised subjects, *Pneumocystis jirovecii*, the species infecting human beings, provokes a severe pneumonia (*Pneumocystis* pneumonia or PcP) that has long been regarded as an AIDS-defining illness [1]. Nowadays, many patients are still at risk for PcP even if the introduction of highly active anti-retroviral therapy (HAART), the improvement of patient care in intensive care unit (ICU) and *Pneumocystis* chemotherapy have reduced the prevalence of PcP [2], [3]. These patients include those who are unaware of their HIV-status, who have no access to HAART or who are intolerant or non-adherent to treatment. In addition, PcP continues to be of great concern in immunocompromised HIV-negative individuals such as patients with primary immunodeficiency, patients receiving immunosuppressive therapies for malignancies, organ transplantations or autoimmune diseases [4].

Next to clinically prominent PcP occurring in immunocompromised patients, the growing impact of more silent carriage of low burden of *Pneumocystis* organisms in the lungs of non-severely-immunocompromised subjects is now being recognized. Indeed, immunocompetent individuals could be colonized by the fungus and could act as carriers [5], although clinical data are still controversial to clearly establish aerial transmission to susceptible hosts [6], [7]. In animal models, *Pneumocystis* organisms are able to replicate in the lungs of immunocompetent hosts and can be transmitted by airborne route to susceptible hosts via a second immunocompetent host [8]. Moreover, the role of *P. jirovecii* as co-morbidity factor in chronic pulmonary diseases such as chronic obstructive pulmonary disease has recently been reported [2], [9].

The fungi of the genus *Pneumocystis* are now being classified within the Taphrinomycotina subphylum, an early-diverging lineage of Ascomycetes [10]–[12]. Although their taxonomical position has become clearer over the years, uncertainties regarding how these organisms proliferate, have led several authors to draw diverse hypothetical life cycle schemes (reviewed in [13]). Clarification of the missing links in the life cycle would come from a robust and long-term culture model that would enable the dynamic follow-up of *Pneumocystis* stage differentiation and maybe the set up of mating experiments. Hitherto, no such a model is available and hypotheses on the *Pneumocystis* life cycle have to rely on data from experimental pneumocystosis in laboratory animals, transmission electron microscopy (TEM), computer-aided three-dimensional reconstructions as well as recent molecular studies (reviewed in [14]).

Another step further towards the understanding of the life cycle is the possibility of using the fungal species infecting the rat, *Pneumocystis carinii*, and sorting its life cycle stages by flow cytometry in order to study their transmission efficiency, their transcriptome or their ploidy level [15]. Yamada *et al*. [16] were the first to compare the relative amount of nuclear DNA in 4′, 6-diamidino-2-phenylindole (DAPI)-stained *P. carinii* cyst and trophic forms using epifluorescent microfluorometry. The total amount of nuclear DNA in a cyst containing 8 spores was approximately 8 times that of a small-type trophic form [16]. When examining the nuclear DNA content of trophic forms, two peaks of fluorescence intensities corresponding to one content (1C) and two contents (2C) of DNA were visible. Gradual transition between 1C and 2C of DNA was apparent. Few nuclei with multiple DNA contents were also noticed [16]. Later, haploidy was suggested from gene mapping experiments on electrophoretic karyotypes of whole *Pneumocystis* population [17]. Fifteen gene-specific DNA probes were hybridized to individual bands on 8 karyotype profiles that were produced by pulse-field gel electrophoresis [18], [19]. Strengthening this view, quantitative image analysis of *Pneumocystis* individual nuclei stained with fluorescent dyes revealed that both trophic forms and spores were haploid [20]. Nevertheless, Hong *et al*. [21] noticed a split of chromosomes in 2 less intensive bands while studying karyotype variability existing among *P. carinii* and *P. jirovecii*. They postulated diploidy to be and explanation for such a split because homologous chromosomes could be of different sizes. In 2002, Cornillot *et al*. [22] also identified homologues for at least two *Pneumocystis* chromosomes on two-dimensional pulse-field gel electrophoregrams, thus raising some questions about the ploidy level of the *Pneumocystis* nucleus.

In the present work, the DNA contents of sorted pure trophic and cystic forms of *P. carinii* was measured by flow cytometry and compared with those of *Saccharomyces cerevisiae* haploid and diploid strains. Data from this analysis indicate that trophic forms are haploid in majority but also include cells with two, three and four nuclear DNA contents. Not surprisingly, cystic forms displayed eight contents of DNA. The most widely accepted hypothesis on the *P. carinii* life cycle [13], [23], [24] could be strengthened and refined by ploidy analysis of its life cycle stages.

## Results

**Figure 1:**
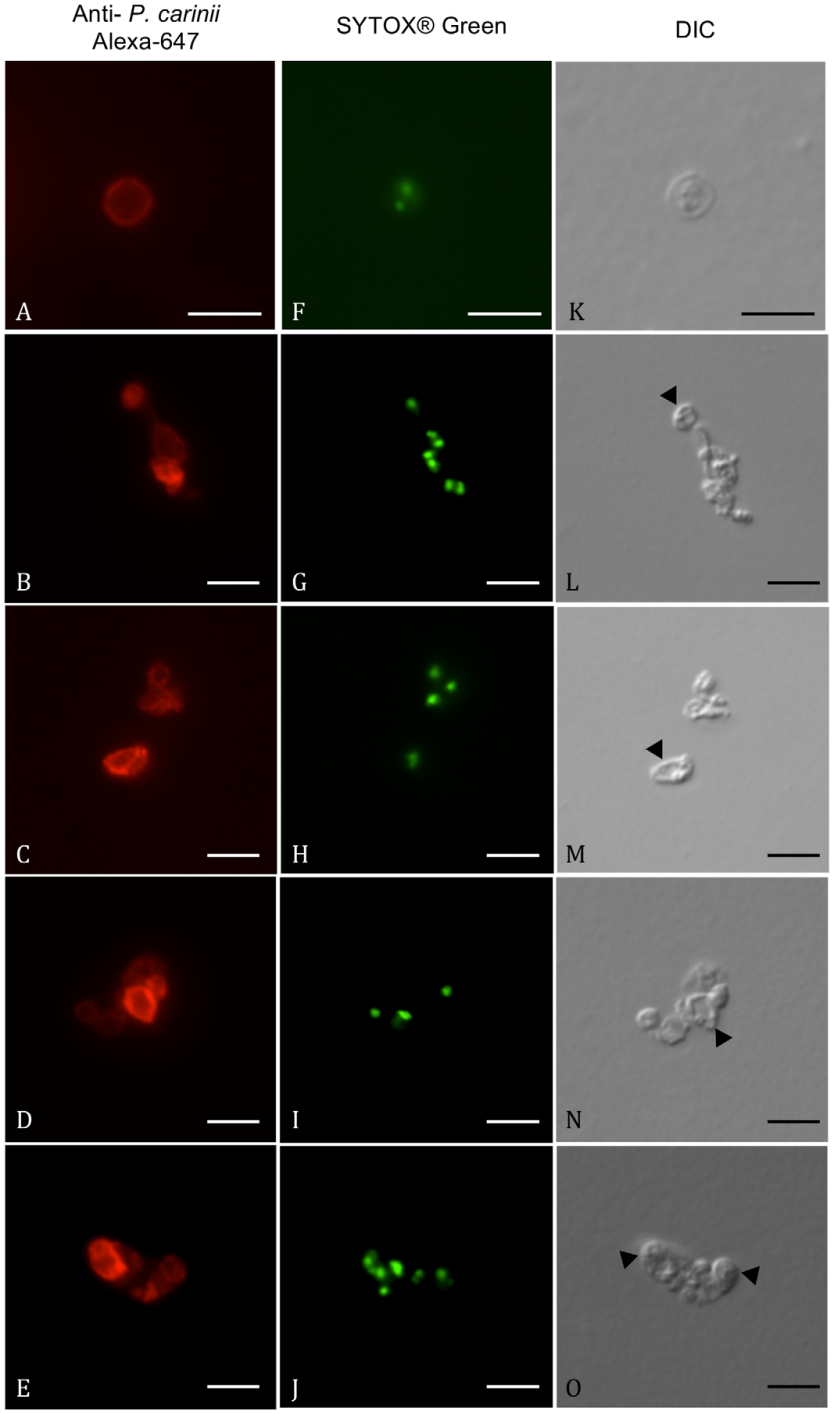
SYTOX® Green labeling of nuclei of *P. carinii* trophic and cystic forms. Home-made polyclonal antibody, recognized by Alexa-647 conjugated secondary antibody, labeled all *P. carinii* life cycle stages in red (A–E). Corresponding fields showed SYTOX® Green discrete nuclear staining (F–J). Differential Interference Contrast (DIC) pictures of corresponding fields were shown (K–O). Panels A, F, K, showed an isolated cystic form with two labeled nuclei. In the other panels, cystic forms (thick-walled stages, arrowheads) and trophic forms (thin-walled stages) are well visible. Bar = 5 µm.

In order to measure DNA content of either *P. carinii* trophic or cystic forms, cell sorting of each stage population was required. Prior to the sorting step, co-labeling using both a polyclonal antibody recognizing all *Pneumocystis* forms as well as a cyst-specific monoclonal antibody was performed [15]. Secondary Alexa-647-conjugated antibody was adequately reacted with corresponding primary antibody depending on the targeted *P. carinii* fraction (either trophic or cystic forms) that was to be incubated with SYTOX® Green. This DNA intercalating agent has been shown to substantially improve cell cycle analysis when compared with conventional DNA binding dyes such as propidium iodide [25]. The figure 1 shows ethanol-fixed and RNase-treated *P. carinii* organisms that were labeled with an anti-*P. carinii* polyclonal antibody (red) and stained with SYTOX® Green. Discrete nuclear staining of both *P. carinii* trophic and cystic forms enabled subsequent cell cycle analysis.

**Figure 2:**
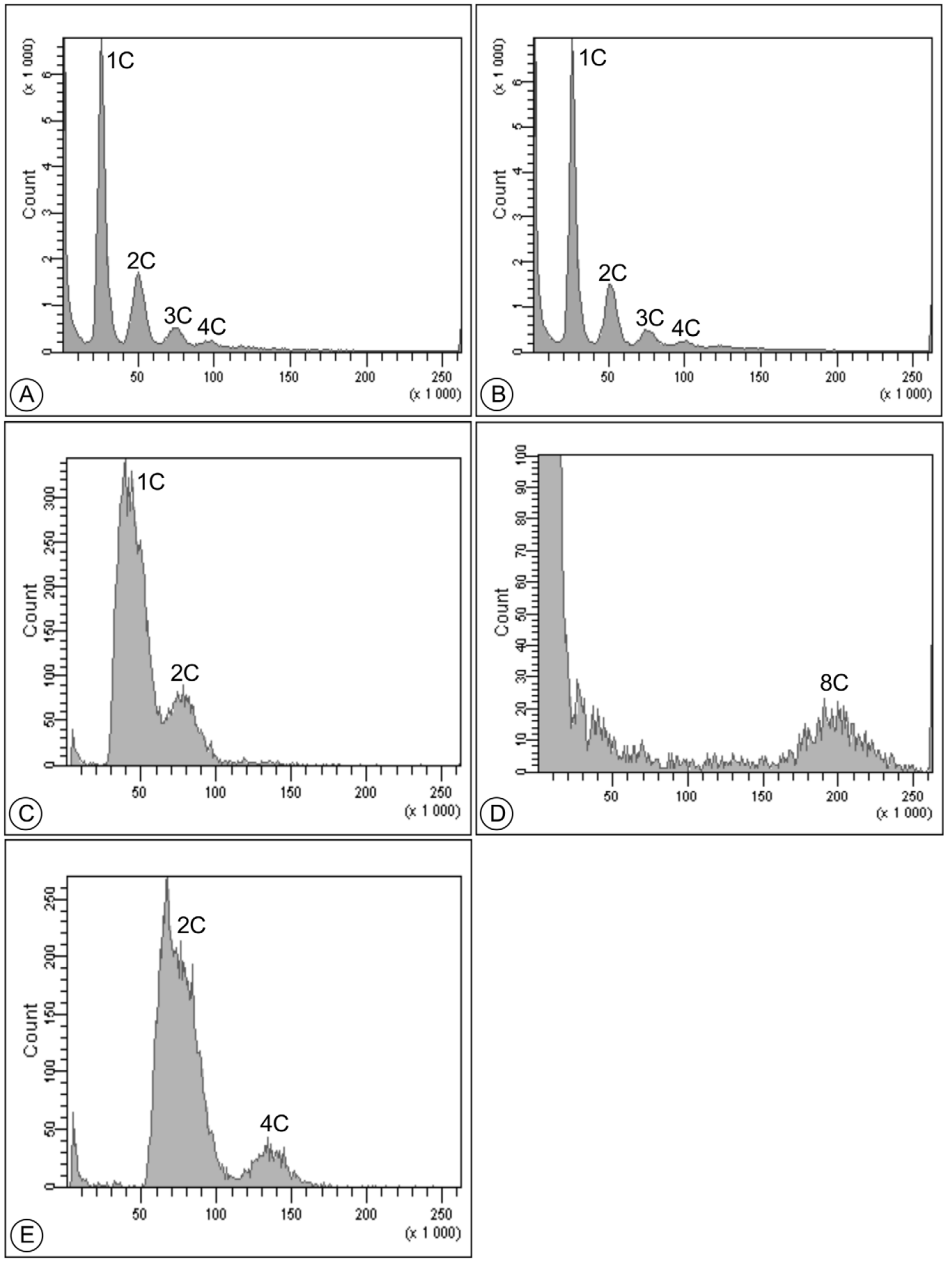
Representative cell cycle analysis histograms of sorted *P. carinii* organisms. DNA contents of (A) all sorted *P. carinii* life cycle stages; (B) sorted *P. carinii* trophic forms; (C) haploid *S. cerevisiae* reference strain; (D) sorted *P. carinii* cystic forms; (E) diploid *S. cerevisiae* reference strain. For all histograms, the number of cells (Count, Y-axis) was plotted against the fluorescence intensity of DNA-bound SYTOX® Green (channel number (area), X-axis). Cell acquisition was done as follows: (A) and (B) 100,000 cells; (D) 20,000 cells; (C) and (E) 10,000 cells.

To determine the average DNA content per cell, *P. carinii* cells were analyzed by flow cytometry (Fig. 2). Starting from different pools of *P. carinii*, thirteen independent experiments were performed giving reproducible DNA profiles. Fluorescence of DNA-bound SYTOX® Green is proportional to the quantity of DNA contained in each cell nucleus. Four peaks that corresponded respectively to 1C, 2C, 3C and 4C of DNA distinctly appeared within the whole *P. carinii* population as well as in the trophic form fraction (Fig. 2A and B). Thus, whole *P. carinii* and trophic form populations showed comparable DNA content profiles. Cells with 1C of DNA were the most abundant: 64.6% in the total population and 63.7% in the trophic forms. Cells with 2C of DNA represented 22.5% in the fraction containing all life cycle parasite stages and 23.9% in the trophic form fraction. Cells with 3C of DNA represented 8.4% in both fractions. Cells with 4C were the least abundant with 4.5% and 4.0% in the total population and in the trophic form fraction, respectively.

Reference haploid and diploid strains of *Saccharomyces cerevisiae* grown to exponential phase were also included in the study as external standards. In the haploid strain, two peaks corresponding to 1C and 2C of DNA were displayed (Fig. 2C) while peaks corresponding to 2C and 4C of DNA were got in the diploid strain (Fig. 2E). The data of DNA contents of yeast and *P. carinii* cells were acquired at the same voltage in order to be able to compare fluorescence intensities (Table 1). As the genome size of *S. cerevisiae* (12.1 Mb, [26]) is larger than that of *P. carinii* (8 Mb, [17]), yeast fluorescence intensity values appear slightly higher than those of *P. carinii* when comparing corresponding peaks (i.e. 1C of *P. carinii* to 1C of *S. cerevisiae*). When data were normalized according to genome size difference, the intensity values of 1C, 2C and 4C peaks of *P. carinii* were similar to the corresponding values of *S. cerevisiae* haploid and diploid strains (Table 1).

**Table 1:** Mean channel numbers of DNA peaks.

Cell fractions	Sub-fractions according to DNA content				
	1C	2C	3C	4C	8C
All *Pc* life cycle stages(Fig. 2A)	25.8 (9.1)*39.0*	49.9 (5.2)*75.5*	74.2 (4.2)	97.5 (3.3)*147.5*	N. a.
*Pc* trophic forms (Fig. 2B)	25.9 (7.9)*39.2*	51.1 (6.1)*77.3*	75.6 (4.4)	100.1 (4.0)*151.4*	N. a.
*Pc* cystic forms (Fig. 2D)	N. a.	N. a.	N. a.	N. a.	198.5 (5.2)
Haploid *Sc* strain(Fig. 2C)	41.1 (9.4)	77.5 (6.2)	N. a.	N. a.	N. a.
Diploid *Sc* strain(Fig. 2E)	N. a.	71.8 (9.5)	N. a.	135.8 (3.8)	N. a.

Mean channel numbers of DNA peaks of sorted *Pneumocystis* fractions (all *P. carinii* life cycle stages, trophic and cystic forms) were compared to those of haploid and diploid strains of *Saccharomyces cerevisiae*. Coefficients of variation are indicated between brackets and are determined at half the height of the peak. When useful, fluorescence intensity values of *P. carinii* have been normalized for genome size difference (numbers in *italic*) in order to facilitate comparison with the yeast values. The ratio between *S. cerevisiae* and *P. carinii* genome size is 1.5 [26], [17]. *Pc*: *P. carinii*. C: DNA content(s). N. a.: Non applicable. *Sc*: *S. cerevisiae*.

A single peak of 8C of DNA characterized the cystic form fraction (Fig. 2D). However, this fraction included also intermediate and late sporocytes. In order to check for the proportion of the 1-to-8 nucleated sporocytes and mature cysts in the cystic form fraction, *P. carinii* organisms were isolated from rat lungs as previously described [27]. RAL-555-stained parasites were microscopically quantified according to the number of nuclei they contain (Table 2). Late sporocytes and mature cysts with 5 to 8 nuclei represented above 71% of the whole cystic form population. A smaller proportion (close to 27%) consisted of intermediate sporocytes containing 2 to 4 nuclei while the early sporocytes with a single nucleus were in minority (Table 2).

**Table 2:** Proportions of sporocyte and mature cyst stages of *Pneumocystis carinii*.

N	Mean % of fungal cells with N nuclei ± SD	Mean % of fungal cells with N nuclei ± SD
**1**	1.43±0.31	**1.43±0.31** (a)
**2**	8.10±2.52	**26.93±1.39** (b)
**3**	3.07±1.54	
**4**	15.77±3.74	
**5**	11.50±4.09	**71.64±1.25** (c)
**6**	9.40±2.04	
**7**	14,60±2.69	
**8**	36.13±6.72	

Whole organism population was isolated from the lungs of immunosuppressed animals as previously described [27]. Sporocyte and mature cyst stages were counted from RAL-555-stained smears according to the number of nuclei in each life cycle stage. Percentages of sporocyte and cystic stages with N nuclei in the fraction of sporocytes and mature cysts were given as mean of three independent experiments (n = 70, n = 55, n = 50). N = number of nuclei per fungal cell. SD = standard deviation.

(a) Early sporocytes.

(b) Sum of intermediate sporocytes with 2, 3 and 4 nuclei.

(c) Sum of late sporocytes and mature cysts with 5, 6, 7 and 8 nuclei.

**Figure 3:**
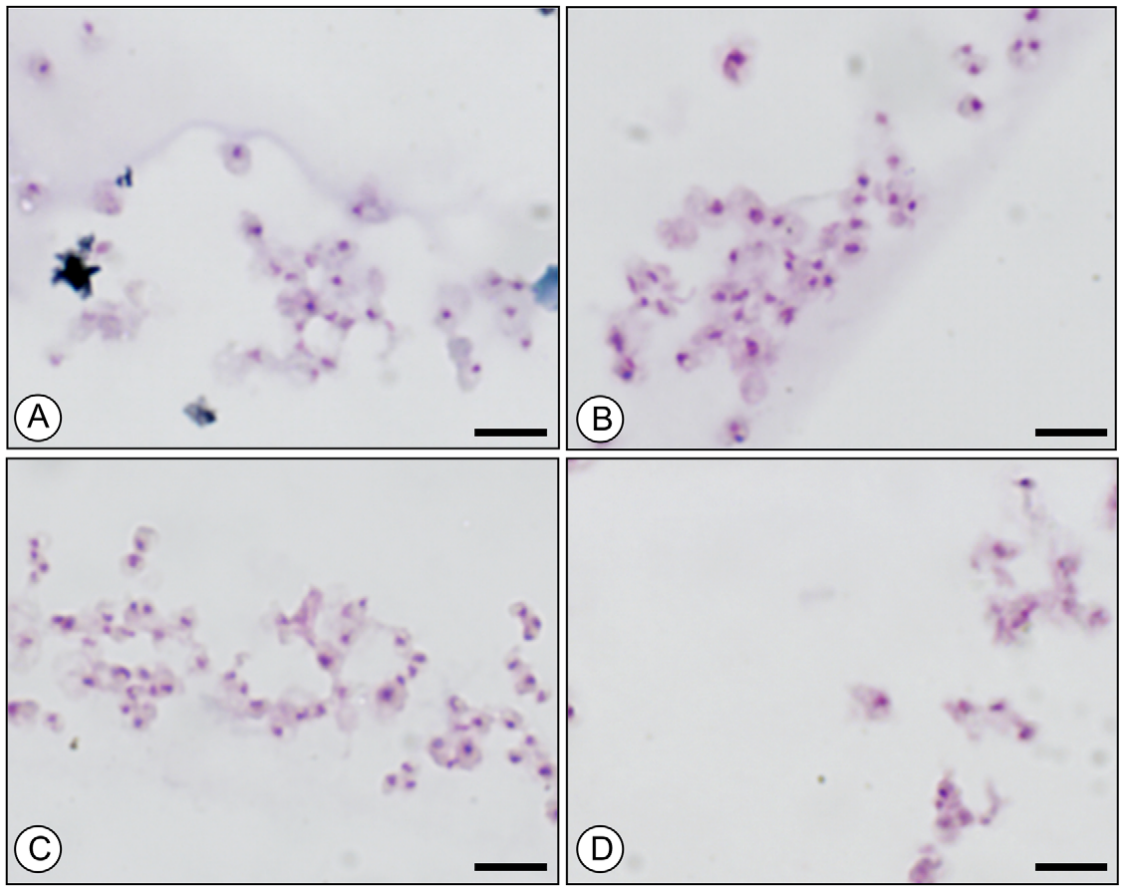
Sorting of *P. carinii* organisms according to their DNA contents. RAL-555 staining of *P. carinii* organisms containing one (A), two (B), three (C) or four contents of DNA (D). Bar = 10 µm.

**Figure 4:**
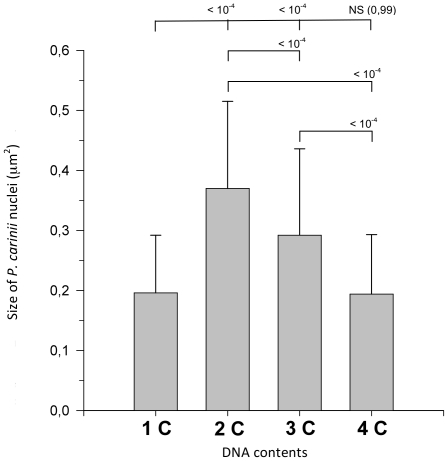
Histogram representing mean areas of nuclei of *P. carinii* organisms according to their DNA contents. Following cell sorting according to *P. carinii* DNA content, images of Giemsa-like stained *P. carinii* organisms were analysed using the automated ImageJ software. Statistical analysis using F-test and Tukey test was performed. The p-values are indicated for each pairwise comparison. A p-value<0.05 is deemed to be significant. NS = non significant.

The *P. carinii* total population was sorted according to the DNA content of each cell in order to explore potential relationships between specific morphological trait of *P. carinii* cells and their DNA content. Images of *P. carinii* organisms containing 1C, 2C, 3C and 4C of DNA were displayed on Figure 3. No obvious morphological differences (either with respect to the aspect of cytoplasm or nucleus) were noticed between organisms with 1C, 3C and 4C of DNA (Fig. 3A, C and D, respectively). However, the size of *P. carinii* nuclei containing 2C of DNA (Fig. 3B) appeared enlarged compared to those with 1C, 3C or 4C of DNA. This was confirmed by the automated image analysis driven by the ImageJ software (Fig. 4). Indeed, the mean area of nuclei of trophic forms with two contents of DNA were 1.9 times larger than that of nuclei of trophic forms with one content of DNA (p<10^−4^). The mean area of nuclei significantly decreased when comparing nuclei with 2C *versus* 3C and 3C *versus* 4C of DNA (p<10^−4^ for each pairwise comparison). The mean area of nuclei with 4C of DNA was comparable to that of nuclei with 1C of DNA. Indeed, the area size difference between 1C and 4C nuclei was statistically non significant (p = 0.99).

**Figure 5:**
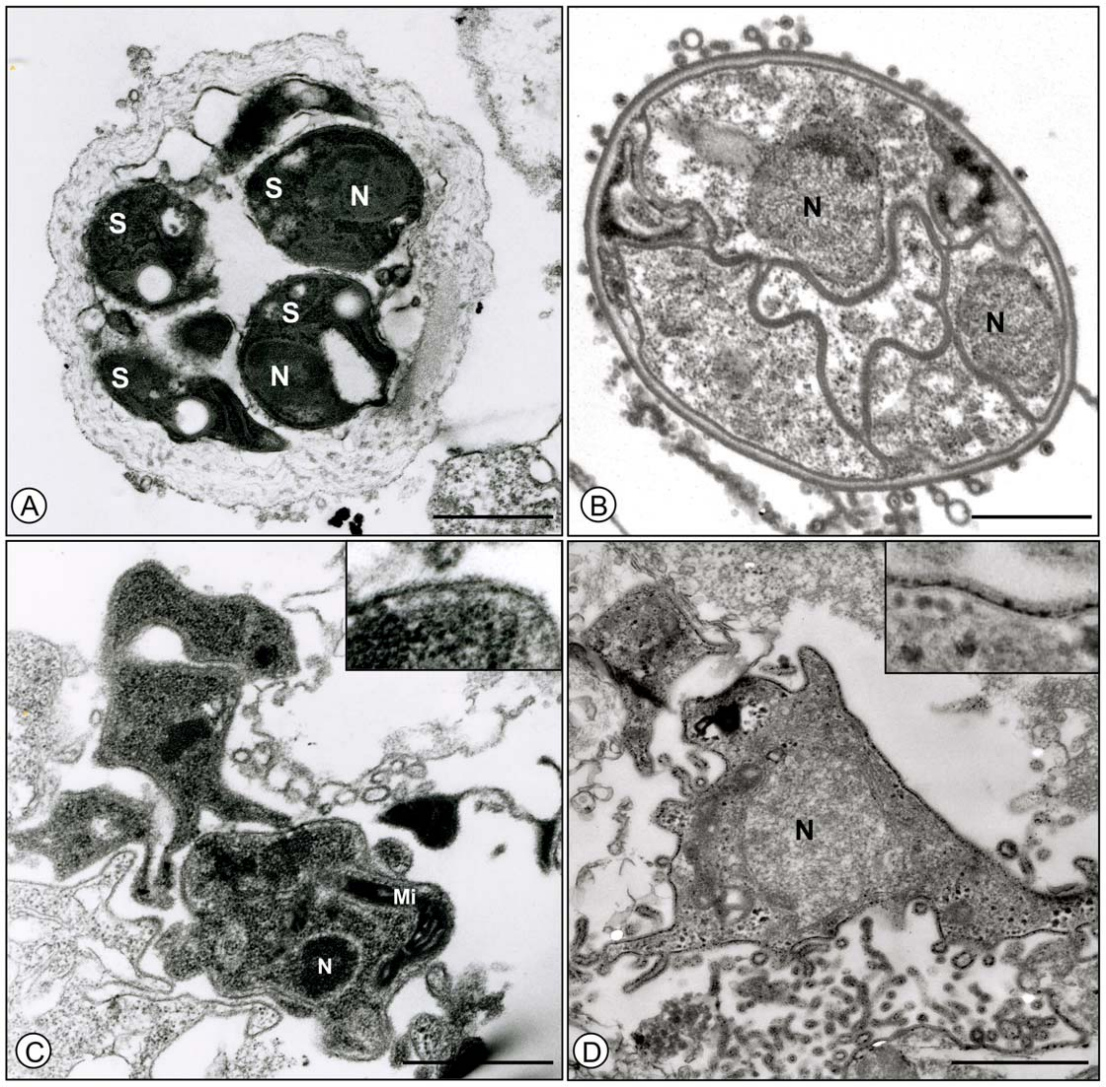
Transmission electronic microscopy of untreated or sorted parasite stages. (A) A *P. carinii* sorted cystic form; (B) an untreated intermediate sporocyte : the thick cell wall and two nuclei are well visible; (C) a *P. carinii* sorted trophic form; (D) an untreated trophic form. Trypsin treatment, required for antibody labeling of the sorted cystic forms, altered their cell wall external dense layer. The electron dense layer of the sorted trophic form cell wall was also altered but not completely removed while the plasma membrane remained intact (see insets C and D). Cell wall lucent layers of cystic forms, as well as cytoplasmic or nuclear structures of both life-cycle stages, were ultrastructurally unaltered. Bar = 1 µm. N = Nucleus, Mi = Mitochondrion, S = Spore.

To check for ultrastructural integrity, sorted trophic and cystic forms of *P. carinii* were observed using TEM (Fig. 5). Sections of four well-preserved spores bearing intact nuclei were clearly visible within a mature sorted cyst form in Figure 5A. The cyst cell wall has been affected by the immunostaining procedure [15] thus leading to the removal of electron-dense layer and expansion of the electron lucent layer as compared with an intact intermediate sporocyte (Fig. 5B). The electron dense layer of the sorted trophic form cell wall (Fig. 5C) appeared thinner than that of untreated trophic form (Fig. 5D); but it was not completely removed and the plasma membrane remained intact (Fig. 5C). Well-preserved organelles such as mitochondria and nuclei were easily recognized either in sorted (Fig. 5A, C) or untreated life cycle stages (Fig. 5B, D).

## Discussion

Flow cytometry enables to analyze multiple parameters of a high number of individual cells thus reaching statistical significance [28]. So far, authors interested in the ploidy level of *Pneumocystis carinii* have either (i) undertaken a population based approach looking at chromosomes of an entire *P. carinii* population [18], [19], [21], [22] or (ii) analyzed cell by cell DNA content by using a low throughput quantitative fluorescence image analysis [16], [20]. In our approach, taking advantage of the multi-parameter strategy available in high throughput flow cytometry, it was possible to physically separate stage populations of *P. carinii* and analyze DNA contents of up to 100,000 trophic forms and 20,000 cystic forms.

From the DNA content analysis, it appears that the major part (64%) of *P. carinii* organisms isolated from nude rats with extensive PcP, contains 1C of DNA. Once normalized for the genome size difference existing between both fungal organisms, the fluorescence intensity of *P. carinii* 1C peak was comparable to the 1C peak of *S. cerevisiae* haploid strain (Fig. 2) thus suggesting that most of *P. carinii* organisms were haploid. In 1998, Wyder *et al*. [20] also reported that *P. carinii* organisms were haploid from quantitative microscopic fluorescence analysis of DNA contents. On the contrary, Yamada *et al*. [16] argued for a bimodal distribution of DNA contents in trophic forms using a similar approach. A majority of those forms bore 1C of DNA while a sub-population contained 2C of DNA [16]. Nevertheless, Wyder *et al*. [20] argued that artifactual staining from cell wall could not be ruled out, since *P. carinii* organisms had not been treated with lyticase [16]. As specific immunostaining of cystic forms requires moderate trypsin digest to reveal the epitope, partial removal of *P. carinii* cell wall prevented artifactual binding of SYTOX Green® in our experiment (Fig. 1), thus leading to a positive correlation between *P. carinii* DNA content and SYTOX Green® fluorescence. Nonetheless, we could not rule out the staining of mitochondrial DNA by the dye. As mitochondrial DNA is small (23 kb, [29]), its relative contribution to nuclear fluorescence intensity remains negligible. Moreover, computer-aided three-dimensional reconstruction showed that a unique mitochondrion is present in the studied life cycle parasite stages [30], [31], thus keeping the ratio mitochondrial-to-nuclear DNA content to one in these forms. It is however conceivable that the mitochondrion starts budding and replicating DNA at the intermediate sporocyte stage in order to produce one mitochondrion per spore in the mature cysts [32]. Because nuclear DNA is also replicating during the sporocytic maturation, mitochondrial material will most probably never reach enough relative abundance to significantly affect quantitation of nuclear DNA contents.

The DNA content analysis of total *P. carinii* population reveals that cells containing 1C of DNA represent the main but not the sole population in agreement with Yamada *et al*. [16]. Life cycle forms of *P. carinii* harboring 2C, 3C and 4C of DNA were also present but in decreasing proportions (Fig. 2A). It is well probable that the high number of individual cells analyzed in our study allowed to reveal less abundant sub-fractions (3C and 4C), which could not be detected using traditional microscopic approaches because much less cells could be analyzed [16], [20].

In the sorted *P. carinii* cystic form fraction, eight contents of DNA could be measured (Fig. 2D, Table 1). As it is known that one mature cyst contains eight uninucleated haploid spores [32], these data imply that each spore lying within a mature cyst contains one DNA content as it occurred in the most abundant sub-fraction of trophic forms. This finding is in agreement with the report by Wyder *et al*. [20] where they claimed that nuclei of trophic and cystic forms essentially contained equivalent amounts of DNA.

DNA content profiles are similar in the *P. carinii* trophic form fraction and in the total population although the 8C peak should theoretically become apparent in the latter (Fig. 2A and B). Nevertheless, as expected in the lungs of hosts developing extensive PcP, cystic forms only represent 1 to 2% of the total population and their scarcity prevents distinct visualization of the corresponding DNA content peak among the *P. carinii* total population. Thus, sorting is a prerequisite to be able to analyze DNA content of cystic forms (Fig. 2D).

The monoclonal antibody used to sort the cystic form fraction recognizes an epitope embedded within the electron-lucent layer of the cell wall [15], [33]. This β-glucan rich layer starts to be deposited at the intermediate sporocyte stage and thickens at the late sporocyte and mature cyst stages. It is then expected that these stages segregated within the sorted *P. carinii* cystic form fraction. Consequently, the distribution of DNA contents in this fraction should be bimodal with a 4C peak corresponding to intermediate sporocytes and 8C peak corresponding to late sporocyte and mature cysts. Yet, *P. carinii* cystic forms containing four contents of DNA do not appear in our analysis (Fig. 2D). Two combined reasons may explain this finding: (i) it is conceivable that too few cells with 4 contents of DNA are present in the cystic form fraction to be detected by the flow cytometer. Indeed, counting the cystic forms with 1 to 8 nuclei on RAL-555-stained smears made from an unsorted total *P. carinii* population isolated from rat lungs (Table 2) revealed that late sporocytes and mature cysts (5 to 8 nuclei) were the most abundant parasite stages among the forms with a thick cell wall. Intermediate sporocytes which are likely to contain four contents of DNA only represent close to 27% of the total cystic form population, which accounts, amongst the *P. carinii* total population, for fairly few cells (<1%); (ii) while measuring fluorescence intensity from the cyst-specific antibody, the sorting gates have been set to avoid contamination of trophic forms within cystic forms and *vice versa* [15]. Consequently, a small gap of non-sorted cells exists between these gates. In other words, *P. carinii* cells displaying low-to-medium fluorescence (i.e. intermediate sporocytes) could be lost from our analysis.

**Figure 6:**
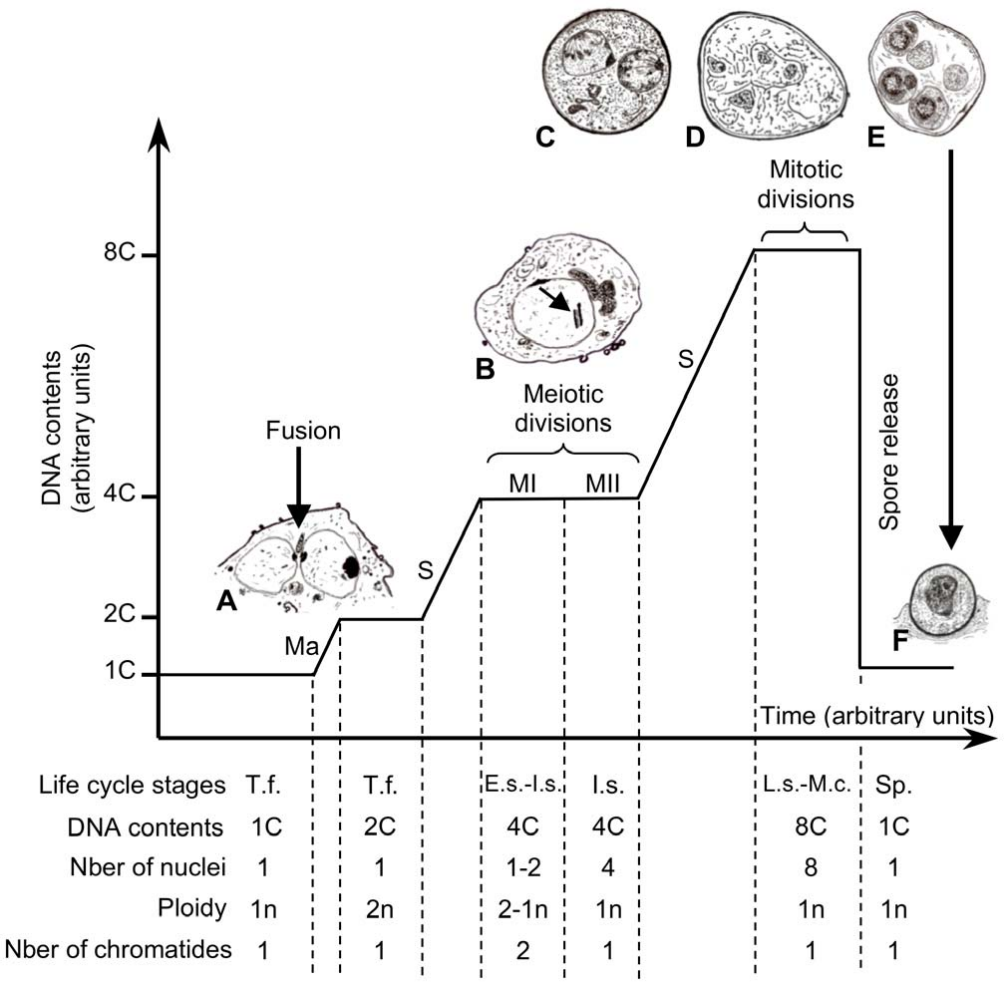
Interpretation scheme of *P. carinii* life cycle and cell cycle. Two haploid trophic forms (T.f.) mate (Ma) to produce a zygote with an enlarged single diploid nuclei. DNA synthesis (S) occurs to give rise to diploid early sporocytes (E.s.) which undergo the first meiotic division (MI), subsequently leading to intermediate sporocytes (I.s.) with 2 haploid nuclei each containing one set of chromosomes with two chromatides per chromosome. The second meiotic division leads to the separation of chromatides in four haploid nuclei within each intermediate sporocyte. DNA content (4C) does not change during meiotic divisions. A final mitosis produces late sporocytes (L.s.) and mature cysts (M.c.) with 8 contents of DNA shared between the 8 nuclei. Once fully matured, the cysts release 8 haploid spores (Sp.). Ploidy values (n) indicate the number of chromosome sets per nucleus. Time laps of each phase of the *P. carinii* life cycle are arbitrary. (A) Nuclear fusion of two trophic forms. Spindle pole bodies are clearly visible. Drawn according to [58]. (B) Early sporocyte in which a synaptonemal complex is indicated (arrow). Drawn according to [40]. (C) Intermediate sporocyte. (D) Late sporocyte. (E) Mature cyst. (F) Released spore.

Analysis of DNA contents of the trophic form fraction also reveals the existence of cells with two and four amounts of DNA (Fig. 2B). Haploid trophic forms most probably undergo mating or fusion (Fig. 6) thus giving rise to diploid zygote with an enlarged nucleus (Fig. 4). Mitotically dividing trophic forms most probably also contribute to the 2C peak. Some authors have suggested that an asexual cycle involving trophic forms may occur during *Pneumocystis* growth [34]–[36]. Nevertheless, TEM pictures showing trophic forms in a process of mating or binary fission are difficult to interpret and infrequently reported [32], [37], which is surprising giving that these forms represent 98% of the total fungal population. Though, to decipher between fusion and division of trophic forms remains a challenge [38]–[40]. Furthermore, using differential parasite stage counting, Aliouat *et al.* [37] showed *in vitro* that the increase in the number of *Pneumocystis* cells resulting from cyst development and sporogenesis was enough to explain the normal growth of *P. carinii*.

The 4 contents of DNA measured in trophic form nuclei probably result from the DNA synthesis that takes place just prior the beginning of meiosis (Fig. 6), subsequently leading to early sporocytes that are hardly distinguishable from trophic forms by optical microscopy. Looking at the hypothetic life cycle schemed by Yoshida [24], trophic forms bearing 4 contents of DNA could also result from mitotic divisions once mating has occurred.

To find nuclei of *P. carinii* life cycle stages with 3 contents of DNA was very much striking (Fig. 2). As *P. carinii* organisms are known to form aggregates, extra precautions were taken to avoid clumping during the immunofluorescence and SYTOX® Green labeling protocols. Aggregates were also excluded *in silico* when analyzing the data after cytometer acquisition. Although chances for the 3C peak of being artifactual cannot be completely ruled out, one can assume its biological relevance: DNA content can greatly vary within one species or during the life cycle of an organism [41]. More specifically, genome plasticity of fungal organisms can translate into ploidy variation within one particular species. Diploid, triploid and tetraploid strains of *S. cerevisiae* were found colonizing a hotspot of biodiversity [42]. In the laboratory, mating of haploid and diploid strains produces a triploid strain whose spore fertility is nonetheless reduced [43]. During meiosis of triploid or tetraploid yeast strains, crosses and genetic reassortments can occur between 3 or 4 homologous chromosomes [43]. Moreover, Gerstein *et al*. [44] showed that ploidy of *S. cerevisiae* can evolve with generation time. While strains were grown asexually for 1800 generations, changes in ploidy occurred from haploid to diploid and tetraploid to diploid. Endomitosis may quite easily explain the haploid-to-diploid evolution. Simultaneous loss of one complete set of chromosomes would entail the switch from tetraploid-to-diploid while preserving euploidy of *S. cerevisiae* cells [45]. Random loss of several chromosomes in *Candida albicans* allowed a tetraploid strain to undergo chromosome rearrangement while reducing its ploidy level to near diploid state [46]. This advantageous parasexual cycle is an alternative to meiosis that appears rarely used by *C. albicans* [47]. Multipolar mitotic division [48] occurring in a tetraploid cell may constitute a possible explanation of asymmetric distribution (1∶3) of genetic material. Such a division mechanism may occur in *P. carinii* thus resulting in the asymmetrical segregation of chromosomes and leading to an odd number (3) of DNA content per nucleus.

Ploidy of each stage is a fundamental genetic trait with important cytological, physiological and genomic implications that can advance the understanding of the modalities of *P. carinii* proliferation. Thus, we propose to refine the hypothesis on the *P. carinii* life cycle by reconciling accepted knowledge we have from cellular studies of the various life cycle stages with ploidy data (Fig. 6). Haploid trophic forms are either able to divide mitotically and/or to mate (Fig. 6A) and, subsequently, produce a zygote bearing a single diploid and enlarged nuclei. The doubled nuclei area of diploid trophic forms is in favor of the occurrence of a nuclear fusion event (Fig. 4). Once trophic forms have mated and DNA synthesis is completed, *P. carinii* nucleus would undergo recondensation as nuclei with 4C of DNA are comparable in size to nuclei containing 1C of DNA (Fig. 4). This phenomenon of nucleus recondensation occurring just prior to meiotic divisions is comparable to what has been observed in *Schizosaccharomyces pombe* [49]. In *P. carinii*, meiotic divisions start at the early sporocyte stage (Fig. 6B) and end at the intermediate sporocyte stage (Fig. 6C). While DNA content (4C) remains stable during meiotic divisions, the number of nuclei contained in the sporocyte increases from 1 to 4 nuclei. Another round of DNA synthesis is needed to reach the late sporocyte stage (Fig. 6D) that contains eight haploid nuclei. Synthesis of cell wall around each nuclei will progressively lead to the formation of eight mature spores enclosed in the mature cyst (Fig. 6E). Upon spore release, the DNA content drops from eight to one in the haploid free spore (Fig. 6F). Attempts to synchronize *P. carinii* cells are required to evaluate time laps that each life cycle stage needs to complete its maturation/differentiation.

## Materials and Methods

### Ethics statement

All animal experiments were performed following the guidelines of the Pasteur Institute of Lille animal study board, which conforms to the Amsterdam Protocol on animal protection and welfare, and Directive 86/609/EEC on the Protection of Animals Used for Experimental and Other Scientific Purposes, updated in the Council of Europe's Appendix A (http://conventions.coe.int/Treaty/EN/Treaties/PDF/123-Arev.pdf). The animal work also complied with the French law (nu 87–848 dated 19-10-1987) and the European Communities Amendment of Cruelty to Animals Act 1976. All experimental protocols involving animals were carried out by qualified personnel. The animal house (accreditation number: A59107, agreement number: B 59-350009) was placed under the direct control of the director of the Pasteur Institute of Lille who is the “designated responsible person” under French law. The study has been approved by the Ethical Committee for experiments on animals of the region Nord-Pas-de-Calais (approval number CEEA 022011).

### Source of *Pneumocystis carinii* organisms

Athymic *Pneumocystis*-free Lou nu/nu rats (Pasteur Institute Lille, France) were used as a source of *Pneumocystis carinii* organisms for all experiments [15]. Briefly, nude rats were administered dexamethasone two weeks prior to non-surgical endotracheal inoculation of cryopreserved *P. carinii* organisms [15]. Inoculation was performed under isoflurane anesthesia, and all efforts were made to minimize suffering. Eight weeks post-inoculation, animals highly infected with *P. carinii* were euthanized and lungs were dissected to isolate the organisms according to the previously described procedure [15]. Every 3 months, 10 immunosuppressed animals were used to isolate a pool of *P. carinii* organisms and to constitute a cryopreserved parasite stock. The final parasite suspension was checked for the presence of any bacterial or fungal contaminants by inoculation on appropriate culture media. Then, parasite organisms were cryopreserved in fetal calf serum (FCS) with 10% dimethyl sulfoxide according to previously published procedure [50]. Cryopreserved *P. carinii* organisms remain infectious for at least nine years [51] and were used in all subsequent experiments.

### *Saccharomyces cerevisiae* reference strains and culture media

*S. cerevisiae* haploid and diploid strains of known genome size were purchased from EUROSCARF (Frankfurt, Germany) and used as reference. The BY4742 haploid strain (Accession number: Y10000; genotype: MATα ; his3Δ 1; leu2Δ 0; lys2Δ 0; ura3Δ 0) and the BY4743 diploid strain (Accession number Y20000; genotype: MATa/MATα his3Δ 0/his3Δ 0; leu2Δ/leu2Δ 0; met15Δ 0/MET15; LYS2/lys2Δ 0; ura3Δ 0/ura3Δ 0) were maintained at 30°C by periodic passages in Petri dishes with YPD (Yeast Peptone Dextrose) solid medium (1% yeast extract, 1% peptone, 2% D-glucose, 2% agar). For DNA content measurements, yeast cells were grown to mid-log phase (OD_600 nm_ = 0.8) in YPD liquid medium at 30°C with aeration on a mechanical shaker (200 rpm).

### Cell sorting of *P. carinii* cystic and trophic forms

*P. carinii* cell suspension was immunostained, as previously described [15], either with one or two antibodies depending on which life cycle stage population was to be analyzed for its DNA content. Single fluorescent immunolabeling was performed (i) either with a rat polyclonal antibody that was produced in our laboratory [15] and that recognizes all stages of *P. carinii* stages (ii) or with a cyst-specific mouse IgM monoclonal antibody that is commercially available (MONOFLUO KIT *P. jirovecii* (*P. carinii*), reference #72738, Bio-Rad, Marnes-la-Coquette, France). Either Alexa-647 goat anti-rat IgG or Alexa-647 goat anti-mouse IgM (Invitrogen Life Sciences, Cergy Pontoise, France) was used as secondary antibodies depending on the primary antibody. These single immunofluorescence stainings were performed in an aim to either sort whole *P. carinii* population or cystic forms. In order to sort trophic forms, double immunofluorescence labeling was required and performed as previously described [15]. Briefly, trypsin-treated parasites were successively incubated for 30 min at 37°C with undiluted cyst-specific commercial monoclonal antibody, with home-made polyclonal antibody and, finally, with an equal volumetric ratio of Alexa-647 goat anti-rat IgG and Alexa-488 goat anti-mouse IgM (Invitrogen Life Sciences, Cergy Pontoise, France) as secondary antibodies. Consequently, cystic forms were labeled in green and red while the trophic forms were labeled in red only. In order to further dissociate parasite clumps, suspended *P. carinii* cells were thoroughly mixed in PBS without Ca^2+^ or Mg^2+^ using a syringe equipped with a 26G needle and filtered through a 50-µm polyester membrane (Filcons, Becton Dickinson, Le-Pont-de-Claix, France) prior to cell sorting.

*P. carinii* life cycle stage populations were sorted using the FACSAria™ cytometer (Becton Dickinson) driven by BD FACSDiva™ software (version 5.0.2, Becton Dickinson) as previously described [15]. The 70-µm nozzle was used for high-pressure sorting (70 psi/4.9 bar). Compensation was not needed because emission spectra did not overlap. A sorting mask (0/32/0) was chosen to prioritize purity versus yield, thus avoiding sorting of two differently labeled events in the same droplet. In order to check for purity, aliquots of sorted cyst and trophic forms were spotted on glass slides to be stained with RAL-555 (Réactifs RAL, Martillac, France), a rapid Giemsa-like stain. *P. carinii* life cycle stages have been named by using the usually accepted nomenclature [32], [52]–[54]. Indeed, the “trophic form” fraction included the parasite stages with a thin cell wall (trophic forms, early sporocytes), while the “cystic form” fraction included the life parasite stages possessing a thick cell wall (intermediate and late sporocytes, and mature cysts).

### Analysis of DNA contents of *P. carinii* sorted life cycle stages

Following cell sorting, reference yeast strains and Alexa-647-labeled fractions of (i) all *P. carinii* forms, (ii) trophic forms or (iii) cystic forms were labeled with SYTOX® Green (Invitrogen Life Sciences, Cergy Pontoise, France), a DNA binding dye, used previously in yeast cell cycle studies [25], [55].

Yeast cells (1×10^7^ cells) and *P. carinii* organisms (7×10^5^ cystic forms, 7×10^7^ trophic forms and 7×10^7^ total *P. carinii* organisms) were harvested by centrifugation at room temperature (2900 *g* for 15 min). After gentle cell suspension in 20 µl of PBS, fixation with 1 mL of ethanol 70% (v/v) is allowed to proceed for an hour at room temperature. Cells were harvested, washed and suspended in 500 µl of sodium citrate buffer (50 mM, pH 7.0), treated for 1 hour at 56°C with RNaseA at a final concentration of 120 µg/mL (Invitrogen Life Sciences, Cergy Pontoise, France) and allowed to cool at room temperature. Just prior analysis, 10 µl of SYTOX® Green (Invitrogen Life Sciences, Cergy Pontoise, France) solution (50 µM SYTOX® Green in 50 mM sodium citrate buffer pH 7.0) was added to 490 µl of *P. carinii* or yeast cell suspension, thus making a final SYTOX® Green concentration of 1 µM. Samples are then mixed with a syringe equipped with a 26G needle and filtered through a 50-µm polyester membrane (Filcons, Becton Dickinson, Le-Pont-de-Claix, France) to avoid cell clumping. The FACSAria™ cytometer (Becton Dickinson) equipped with a Coherent® Sapphire™ solid state 488 nm blue laser was used for analysis of the samples. The photomultiplier E equipped with a standard 502 nm long pass mirror as well as a 530/30 nm band pass filter was used to collect SYTOX® Green fluorescence.

A minimum of 10,000 cells per sample was acquired at a rate of 20,000 events per second and data were analyzed with the BD FACSDiva™ software (version 5.0.2, Becton Dickinson). An acquisition protocol was defined to measure forward scatter, side scatter and far-red fluorescence (Alexa-647) on a logarithmic scale and SYTOX® Green fluorescence on a linear scale. For DNA content analysis, doublets and larger aggregates were discarded by electronic analysis of the width and height of the forward and side scatter signals from the particles analyzed.

### Light microscopic assessment and nuclear size estimate of sorted *P. carinii* organisms

Whole *P. carinii* population was first immunostained with a polyclonal antibody as primary antibody and Alexa-647 goat anti-rat IgG as secondary antibody as described above. Following SYTOX® Green staining, *P. carinii* cells were sorted according to their DNA contents. Gates corresponding to 1C, 2C, 3C and 4C of DNA were set on a histogram plot showing SYTOX® Green fluorescence intensity on a linear scale. Events falling in those four gates were sorted simultaneously using the FACSAria™ cytometer (Becton Dickinson). Sorted *P. carinii* cells were collected by centrifugation and spotted on glass slides to be stained with RAL-555 (Réactifs RAL, Martillac, France). Images were taken for each of the 4 DNA content fractions using an Eclipse E600 microscope (Nikon) equipped with a digital camera (DXM 1200C, Nikon). The free software ImageJ (NIH, USA) was used to measure the size of RAL-555-stained nuclei of *P. carinii*. A short macro was written to automate the image analysis process (same processing steps were applied to every files contained in a directory). As images were taken in RGB format, a separation of the three color components (“Image – Color – Split Channels”) was necessary to obtain a more contrasted image (grey levels - 8 bits). Then, a background subtraction (“Process – Subtract Background” – rolling ball algorithm) followed by a threshold (“Image – Adjust – Threshold”), enabled to eliminate the background signal: only the darkest areas (grey levels between 0 and 205) corresponding to *P. carinii* nuclei were kept. The “Analyse – Analyse Particles” function of ImageJ was used to detect and draw Regions of Interest (ROI) fitted around *P. carinii* nuclei. Only particles showing a size between 0.05 and 1.1 µm^2^ were considered. Aggregates were eliminated from the analysis. Finally, after calibration of pixel size, the ‘Area Measurement’ function (“Analyze – Measure”) calculated the area value for each ROI (corresponding to *P. carinii* nuclei) in µm^2^. Data were statistically analyzed after logit transformation using the PROC MIXED procedure of SAS (SAS Institute, Cary, NC). The model included the ploidy as a fixed effect factor and a REPEATED/GROUP statement to assess the homogeneity of variances among the four groups. Equality of two or more variances was tested with likelihood ratio tests. When the F-test of the ploidy factor showed a significant effect, pairwise comparisons were performed using Tukey tests. Normality of the residuals was evaluated with Shapiro-Wilk tests, normal quantile plots and kernel density plots. A p-value<0.05 was deemed to be statistically significant. Numbers of *P. carinii* nuclei analyzed were 163, 248, 107 and 61 for 1, 2, 3 and 4 contents of DNA, respectively.

### Transmission electron microscopy of sorted cystic and trophic forms of *P. carinii*

Following sorting, fixation and Epon-embedding of *P. carinii* cystic and trophic form fractions were carried out as previously [56] by using high osmolarity fixation and washing solutions that optimized the preservation of cell structures [57]. Ultrathin sections were contrasted with uranyl acetate and lead citrate and examined using a transmission electron microscope (LEO-906, Leica, Rueil-Malmaison, France).
